# Orthodontic brackets friction changes after clinical use: A systematic review

**DOI:** 10.4317/jced.55676

**Published:** 2019-05-01

**Authors:** Sérgio-Elias-Neves Cury, Aron Aliaga-Del Castillo, Arnaldo Pinzan, Karine-Laskos Sakoda, Silvio-Augusto Bellini-Pereira, Guilherme Janson

**Affiliations:** 1 M.Sc., Ph.D. Graduate Student. Department of Orthodontics. Bauru Dental School. University of São Paulo, Brazil; 2 Associate Professor. Department of Orthodontics. Bauru Dental School, University of São Paulo, Brazil; 3 M.Sc. Graduate Student. Department of Orthodontics. Bauru Dental School. University of São Paulo, Brazil; 4 Professor and Head. Department of Orthodontics. Bauru Dental School, University of São Paulo, Brazil

## Abstract

**Background:**

To evaluate the bracket-wire friction force after clinical use.

**Material and Methods:**

A systematic search of several electronic databases (PubMed, Embase, Web of Science, Scopus, The Cochrane Library, Lilacs and Google Scholar) without limitations regarding publication year or language, was performed. *In-vitro* studies analyzing the changes in friction force of orthodontic brackets before/after their clinical use were considered. Risk of Bias was assessed with Downs and Black checklist. All methodological features that could interfere in the results were specifically described.

**Results:**

Seven studies satisfied the inclusion criteria and were included in the review. All 7 studies reported at least two groups (before and after clinical use). Friction force increased after intraoral aging in most of the studies. However, there is lack of good quality evidence in this research area.

**Conclusions:**

Brackets present increased surface roughness after clinical use, and consequently increased coefficient of friction (COF) and Friction Force. Further studies are necessary to obtain more reliable results.

** Key words:**Friction, orthodontic brackets, systematic review.

## Introduction

The sliding resistance of the wire in the bracket slots, during orthodontic mechanics, influences the magnitude of force transmitted to the teeth and may have implications on the efficiency of orthodontic treatment (1). This resistance is directly associated with the friction force of the bracket-wire-ligature system (2), which basically depends on the surface roughness of the system component materials (3,4) and the ligation force used for orthodontic mechanics (5,6).

Intraoral aging of orthodontic materials during clinical use affects their chemical and mechanical properties (7,8). The most common change is increase of the materials surface roughness, which is considered the main cause of bracket-wire friction force increase (8-10). Increase of surface roughness can be caused by: 1) debris and plaque retention (11-13), 2) corrosion due to the presence of bacterial substrate and pH decrease (14,15), 3) scratches performed during professional cleaning with air-powder polishing (16), and 4) frictional interactions between the wire and the bracket slot during sliding mechanics (17,18).

Therefore, understanding of the brackets degradation and how it affects the friction force is essential for clinicians because it could have implications in orthodontic treatment efficiency (19,20).

There are some systematic reviews of *in-vitro* evaluation of the friction force in orthodontic brackets (21,22). Nevertheless, they do not take into account some *in-vivo* bracket characteristics as intraoral aging. Systematic reviews including *in-vitro* studies evaluating brackets friction after intraoral aging (*in-vivo* feature) would bring greater practical information for clinicians. Therefore, this was the motivation for this systematic review.

-Objective

The aim of this systematic review was to assess the available scientific literature including *in-vitro* studies that evaluated the changes in friction force of orthodontic brackets, after their clinical use.

## Material and Methods

-Protocol and registration

The protocol of this systematic review was registered on the International Prospective Register of Systematic Reviews-PROSPERO (CRD42016036275) and is reported according to the Preferred Reporting Items for Systematic Reviews and Meta-Analyses (PRISMA) statement (23).

-Eligibility criteria

The following selection criteria, based on PICOS format, were applied: 1. Participants: Orthodontic retrieved brackets. 2. Intervention/Exposure: Intraoral aging (orthodontic clinical use). 3. Comparison: Orthodontic brackets as received (without orthodontic clinical use). 4. Outcome: Friction Force and Friction Coefficient changes after orthodontic clinical use. 5. Study design: *In-vitro* studies.

Exclusion criteria: Studies without friction force evaluation or performing friction force analysis only before orthodontic treatment, letters to editor, editorials, systematic reviews and meta-analyses.

The search strategy was performed as follows: (orthodontic bracket* OR orthodontic bracket[MeSH Terms] OR orthodontic brackets[MeSH Terms]) AND (retrieved OR retrieval OR received OR aged OR aging OR after) AND (friction* OR friction[MeSH Terms]).

Electronic databases (PubMed, Embase, Web of Science, Lilacs, Scopus and Cochrane Central Register of Controlled Trials) and grey literature search through Google Scholar without limitations regarding publication year or language were performed until November 7th, 2018. Additionally, the evaluators went through the reference lists of the selected articles to ensure that no potential articles were missed.

Two evaluators (S.E.N.C. and A.A.D.C.) independently screened the titles and abstracts identified from the electronic database results after elimination of duplicates. Next, full articles were retrieved to confirm their eligibility. The same evaluators selected the articles for inclusion in the qualitative analysis, independently.

-Data items and collection

The following data were extracted independently by the two reviewers.

Orthodontic Treatment Features: Sample size (number of brackets) bracket types, brand and prescription; wires sequence used in the orthodontic treatment; intraoral aging, referring to the time that the bracket was in the mouth; orthodontic treatment protocol; hygiene standardization; ligature type; and bracket removal.

*In-vitro* Test Features: Brackets storage and cleaning; bracket profile evaluation (before testing); presence or absence of saliva (if *in-vitro* tests were performed in a dry or wet environment); wire section; number of tests per wire; ligature type and force; bracket-wire tipping and torque before (as received), during, and after (retrieved), bracket-wire tipping and torque during the friction test.

-Risk of bias in individual studies

The risk of bias (RoB) of the included studies was assessed using Downs and Black checklist (24), which originally involves 27 questions, and a maximum score of 32 points . However, in the current review, the last item (power assessment, question 27) was simplified by evaluating it as follow: ‘Preliminary power analysis calculation” (yes, 1 point; no or unclear, 0 points), as performed in previous studies (25,26). Therefore, the maximum score for this modified Downs and Black tool was 28, with a higher score indicating Low RoB. Serious methodological limitations were judged to exist when a study collected less than 15 points on the modified scale (27).

Study selection, data collection and the evaluations of RoB in individual studies were independently performed by the two evaluators (S.E.N.C. and A.A.D.C.). Any disagreement was resolved through verbal discussion between the evaluators and with another third evaluator (K.L.S.), when necessary.

Based on the heterogeneity among the studies included in this systematic review, particularly in the way they evaluated the friction force changes after clinical use, it was not plausible to perform a meta-analysis.

## Results

**Figure 1:**
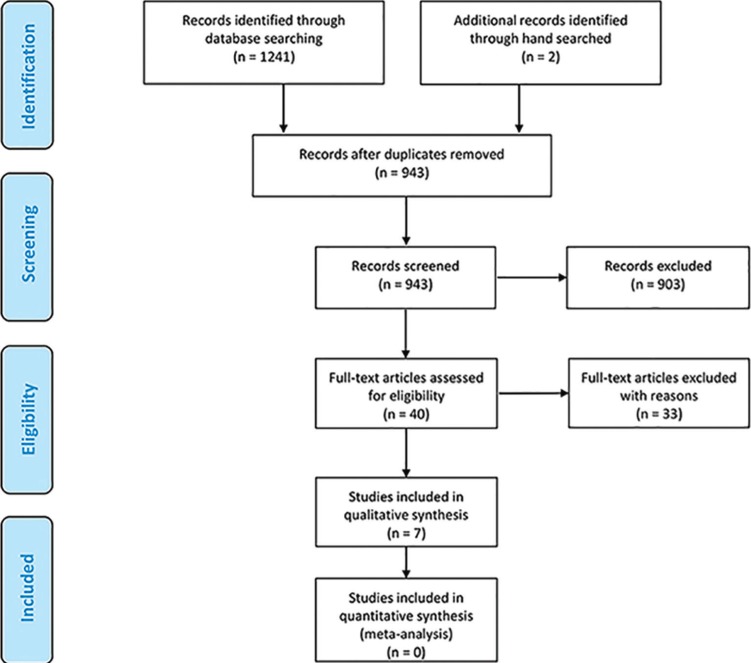
PRISMA Flow Diagram.

Initially, 1241 records were identified, and 2 hand-searched articles were added. After exclusion of duplicates, 943 studies remained. Two evaluators independently screened the titles and abstracts of these articles and 903 were excluded. Then, the full texts of 40 articles were obtained and assessed for eligibility, and 33 articles were excluded for different reasons, leaving 7 articles for qualitative analysis (Fig. 1). All 7 studies that satisfied the inclusion criteria were experimental and included *in-vitro* friction force analyses using as received (without clinical use) and retrieved (after clinical use performing orthodontic mechanics) brackets.

-Orthodontic Treatment Features

Details of all orthodontic treatment features are reported in  Table 1.

**Table 1:**
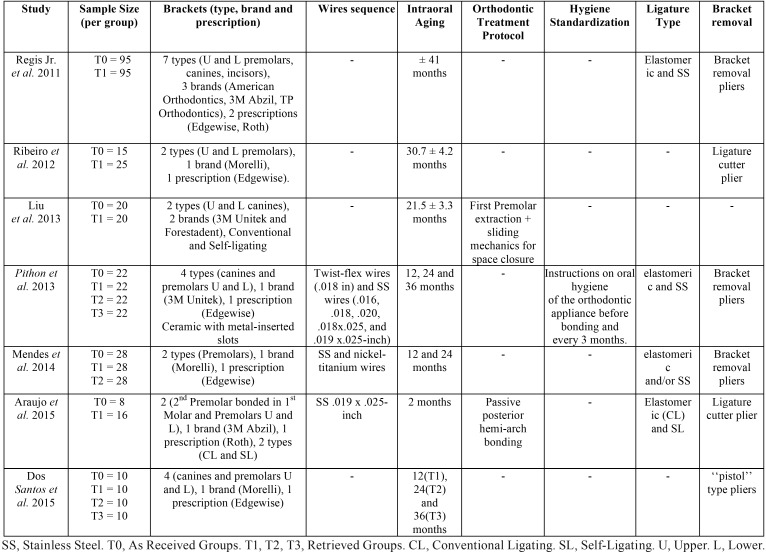
Characteristics of the studies. Orthodontic Treatment Features.

Brackets (type, brand and prescription). With the exception of 1 study (20), which performed *in-vitro* tests with canine brackets, all other trials performed it with premolar brackets (18,28-32). Some studies used brackets of other teeth in addition to the premolar, such as canines (18,30,31), and incisors (31). Four studies (18,29,31,32) used metallic conventional brackets, 2 studies (20,28) used conventional and self-ligating brackets, and one used ceramic with metal-inserted slot brackets (30). Most of the studies (18,29-32) used Edgewise prescription, two studies (28,31) used Roth prescription, and one study did not report the prescription used (20).

Wire Sequence. Only one study (30) mentioned a standardized wire sequence with twist-flex wires and stainless steel (SS) wires. Another study (28) reported the *in-vitro* analysis with the same wire used during intraoral aging. The remaining studies (18,20,29,31,32) did not report the wire sequence during intraoral aging.

Intraoral Aging. Four studies (18,28-30) standardized the time intervals in which the brackets remained in the oral cavity. However, the other three did not used standardization for this issue (20,31,32).

Orthodontic treatment protocol. One study (20) reported performance of first premolar extraction and sliding mechanics used for space closure. Another study used passive posterior hemi-arch bonding (28).

Hygiene Standardization. Only one study (30) standardized the hygiene instructions and protocols in the groups (All patients received instructions on oral hygiene of the orthodontic appliance before installation and every 3 months thereafter).

Ligature Type (During Intraoral Aging). Three studies reported the use of both elastomeric and metallic ligatures (29-31), one study reported the use of elastomeric ligature during intraoral aging (28), and the others (18,20,32) did not report the ligature type.

Bracket removal. Three studies (29-31) used debonding orthodontic pliers for bracket removal. Two studies used ligature cutter plier (28,32). One study used a “pistol” type plier,(18) and another one did not mention the way the brackets were removed (20).

-*In-vitro* Test Features

Details of all *In-vitro* Test Features are reported in  Table 2.

**Table 2:**
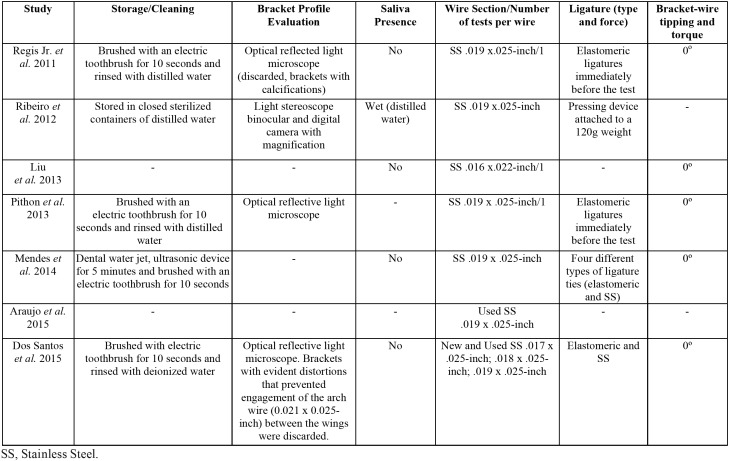
Characteristics of the studies. *In-vitro* Test Features.

Storage/Cleaning. Four articles (18,29-31) reported some type of cleansing of the brackets after removal from the mouth, one study reported no cleaning (32), and two studies did not report anything about bracket storage/cleaning after removal (20,28).

Bracket Profile Evaluation. Four studies (18,30-32) performed the bracket profile evaluation before the friction tests: optical reflected light microscope (18,30,31) and light stereoscope binocular and digital camera with magnification (32). Only two of them reported exclusion of damaged or calcified brackets (18,31).

Saliva Presence/Absence. Only one study (32) performed friction tests in wet environment (distilled water). Four studies performed in dry conditions (18,20,29,31), and 2 studies (28,30) did not report this information.

Wire Section/Number of tests per wire. Except for one study (20), that used a 0.016 x 0.022-inch SS wire to perform the friction force tests, all other selected studies performed it with a 0.019 x 0.025-inch stainless steel wire.

Ligature Type and Force (*In-vitro* Test). Two studies (30,31) performed the friction force test with elastomeric ligatures. Other two studies (18,29) performed it with both elastomeric and metallic ligatures. One study (32) used a system developed by the authors to standardize the ligature force to tie the wire. Two studies (20,28) did not report the ligature type used to perform the friction force tests.

Bracket-wire tipping and torque. Except for one study (28), all of them (18,20,29-32) maintained parallelism between wire and bracket slot, eliminating any tipping between wire and bracket, during the friction force tests. One (20) of them only mentioned that the test specimens were prepared by one experimenter in a standardized way, but the authors did not mention how it was performed. Two studies (30,31) mentioned the use of a holder in a standardized way to guarantee that bracket slots stayed parallel to the testing machine’s vertical axis, but also did not explain how. Other two studies (29,32) only mentioned that care was taken regarding this issue.

-Risk of bias in individual studies

Three (18,28,31) of the seven studies presented medium RoB. Serious RoB were observed in the other four studies (20,29,30,32) and were thus judged as affected by significant RoB ( Table 3). All seven studies had an average score of 13.4 according to the modified Downs and Black checklist.

**Table 3:**
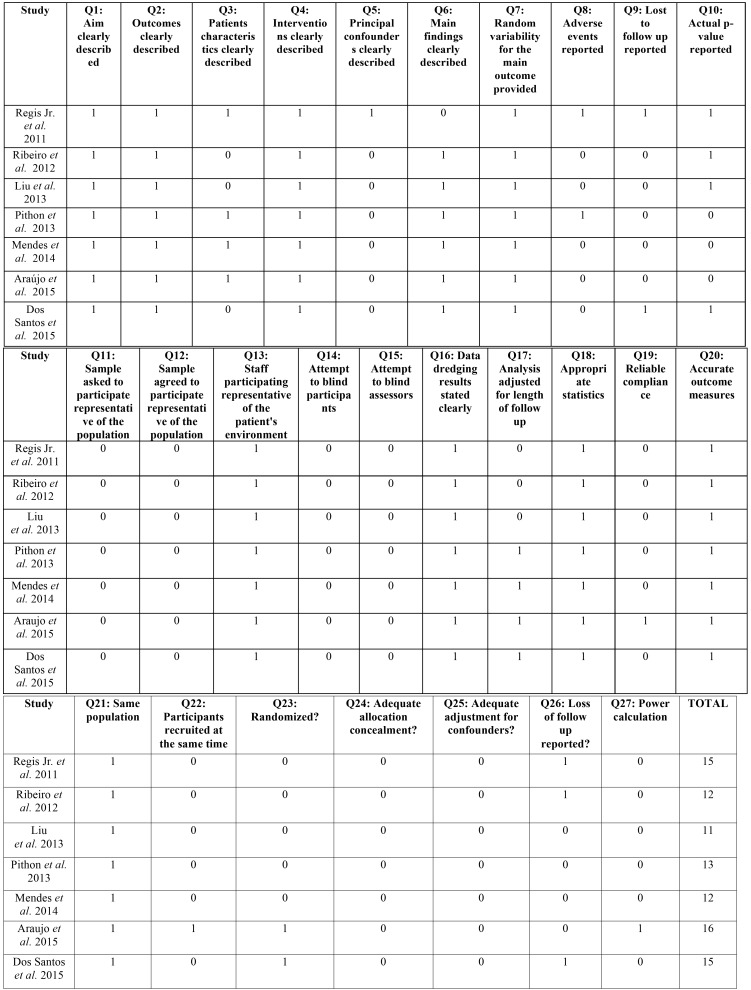
Risk of bias in individual studies.

-Results of individual studies (Main outcome-friction changes)

Six studies (18,20,28,30-32) reported friction force increase after brackets clinical use (retrieved). Only one study (29) reported friction force decrease after brackets clinical use ( Table 4).

**Table 4:**
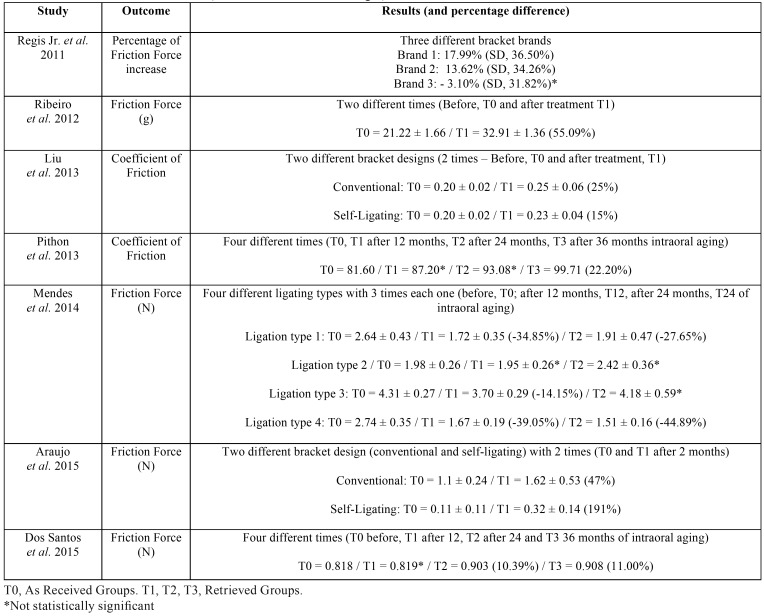
Results of individual studies (Main outcome-friction changes).

## Discussion

Due to the lack of information and consensus about behavior of the intraoral aged brackets during sliding mechanics, this systematic review aimed to evaluate how the aging of brackets during orthodontic treatment impacts on bracket-wire friction and consequently on the sliding mechanics.

This systematic review showed a lack of studies with Low RoB ( Table 3). Four studies (20,29,30,32) had an overall score below the threshold and were judged as affected by significant RoB and three (18,28,31) were judged as affected by some RoB. Additionally, the studies showed heterogeneity in evaluating the changes in friction force because of different friction indicators (Friction Force, Percentage of Friction Force increase and COF). For this reason, a meta-analysis could not be performed.

A qualitative assessment of the studies was performed and the orthodontic treatment (clinical) and *in-vitro* features of the selected studies that could have implications on the evaluation of the friction force after clinical use were separately discussed.

-Orthodontic Treatment Features 

Different types and brands can lead to different behaviors in friction tests (33). Some studies (29,32) included in this review were concerned in using the same type and brand of brackets for friction force evaluation, improving the quality of their results.

The premolar brackets were the most used in the tests as they are the most required for sliding, during retraction mechanics consequent to premolar extraction (28). Therefore, the three studies (28,29,32) included that used only premolar brackets for friction testing present results with direct clinical applications ( Table 1).

Treatment time standardization is important to obtain better friction results. In this review, four studies (18,28-30) standardized the time intervals in which the brackets remained in the oral cavity ( Table 1).

Preventive care with sequential prophylaxis is important to maintain bracket slot surfaces clean and plaque and debris-free (13,15). For this reason, standardization of these procedures are important to avoid sample discrepancies on the friction test results. Unfortunately, this review found that only one study (30) standardized the hygiene instructions in the groups ( Table 1).

It seems that there is no consensus about which bracket design produce smaller plaque and debris accumulation, influencing the friction force test (34,35). Although one study (28) pointed to a greater increase in surface roughness and friction force in retrieved self-ligating brackets compared to retrieved conventional brackets, another study (20) showed no difference between them.

In the current review, the main concern regarding bracket removal was maintaining the integrity of the brackets, which were evaluated *in-vitro*, after removal. Information about the bracket removal method was an important issue to evaluate in the studies, since the integrity of the bracket slots and wings were important for friction force evaluation. Therefore, some studies reported macro and microscopic evaluation of the slots profile before friction assessment, discarding bracket damage (18,30-32), improving the friction tests ( Table 1).

-*In-vitro* Test Features

Two main factors may interfere in surface roughness of the bracket slots and in friction force during orthodontic treatment: debris and scratches (18,28-30). Debris and plaque can be removed during conventional tooth brushing and with professional prophylaxis during appointments (15). Most of the studies included in this review (18,29-31) performed some type of debris removal just before performing *in-vitro* tests. The study that showed a decrease in frictional force was exactly the only one that placed all analyzed brackets in an ultrasound device for 5 minutes before performing *in-vitro* tests,(29) which may be related to the respective results.

There is no consensus about the role of saliva in the friction force during sliding mechanics (36). Therefore, regardless of whether the friction tests were performed in a wet (32) or dry (18,20,29,31) environment, maintaining the same environment during testing is the most important issue.

The 0.019 x 0.025-inch SS wire is commonly used in friction tests because it seems to be the most appropriate wire for space closure when sliding mechanics is used. Smaller diameter wires could produce more binding effects and a greater diameter wire implies in an increase in friction force in almost three times (37).

-Friction Changes Outcomes

Among the seven evaluated studies, only one (29) reported friction force decrease after comparing friction force between as received and retrieved brackets. However, this study (29) may have failed to correctly explain these results, based on scientific evidence. The authors mentioned two articles to confirm this phenomenon, but one of them does not show the results they described (8). Therefore, the controversial results obtained by them may have been a laboratory casualty and has to be understood with caution.

Another study (31) showed a decrease tendency in friction force after clinical use in one sample, but the authors could not explain the reason for this result.

Friction force and COF increased after clinical use in all other studies, (18,20,28,30,32) even in those that performed bracket cleaning before the tests (18,30,31). This was an expected resulted and was reported in previous studies (8,38). It appears to be caused by surface roughness increase, with debris accumulation and scratches, arising by bracket intraoral aging ( Table 4).

-Clinical Implications

Due to the amount of time brackets remain in the mouth during the entire orthodontic treatment, partial loss of the sliding capacity should be considered. It is not possible to measure how much this will influence the mechanics, but in cases of high sliding requirement, it may be advantageous to replace the brackets with new ones, especially the second premolars, which are the ones that will most require sliding of the wire in cases of first premolar extractions.

-Limitations

It was not possible to perform a meta-analysis, since there were several studies judged as affected by significant RoB and due to the heterogeneity among the studies when reporting the main outcome.

## Conclusions

Overall, based on the low to moderate quality evidence, it was found that:

-Brackets present increased surface roughness and consequently, increased COF and Friction Force after clinical use;

-Both conventional and self-ligating brackets are damaged by intraoral aging;

-Further research is necessary to obtain more reliable results.
